# Burden of Chronic Respiratory Diseases (CRD) in Middle East and North Africa (MENA)

**DOI:** 10.1097/1939-4551-4-S1-S6

**Published:** 2011-01-15

**Authors:** F Chermiti Ben Abdallah, S Taktak, A Chtourou, R Mahouachi, Ali Ben Kheder

**Affiliations:** 1 Pneumolgy IV Department, Abderrahmen Mami Hospital, Ariana, Tunisia

**Keywords:** chronic respiratory diseases, chronic obstructive pulmonary disease, asthma, epidemiology, tobacco, burden

## Abstract

Chronic respiratory diseases involve a heterogenous group of diseases, including, chronic obstructive pulmonary disease (COPD), asthma, sleep apnea syndrome, pulmonary hypertension, and many occupational diseases. They affect more than one billion people worldwide. Their medical, social, and economic impacts are heavy, especially in developing countries such as Middle East and North Africa countries, where they represent a public health problem. They are essentially represented by COPD, asthma, and allergic diseases. Chronic respiratory diseases are increasing in frequency, morbidity, and mortality. In addition, their economic and social impact is increasing rapidly in this region. Main risk factors are represented by tobacco smoking and exposure to biomass fuel. Smoking prevention and standardized management programs for asthma and COPD are now available but prompt actions are needed to make them more effective in this region and thus avoid an adverse impact on national economic development.

## Introduction

Chronic respiratory diseases (CRD), essentially chronic obstructive pulmonary disease (COPD), asthma, respiratory allergies, and occupational lung diseases represent a major public health problem in developing countries including the Middle East and North Africa (MENA) countries. This is because of their high frequency and their social and economic impacts. Fortunately, most CRD are preventable and treatable. Several guidelines such as the global initiative for chronic obstructive lung diseases (GOLD), global initiative for asthma (GINA), and allergic rhinitis and its impact on asthma (ARIA) are now very helpful to improve the management of CRD. We try to clarify the burden and trend of CRD and their economic impact in MENA countries from available data in the literature. 

## Prevalence of Main CRD (COPD and ASTHMA) In Mena and Risk Factors

Millions of people of all ages suffer from preventable CRD and respiratory allergies in MENA region. CRD are increasing in prevalence in all ages, but there are considerable differences in prevalence between regions[1].

## COPD

Although COPD is one of the leading causes of mortality and morbidity worldwide, epidemiological data on COPD are very limited, especially in MENA countries. In 1990, the World Health Organization evaluated the global prevalence of COPD to be 9.33 per 1000 for men and 7.33 per 1000 for women[2].

In middle-income countries, such as in MENA, COPD is emerging as public health problem. However, the disease is certainly under diagnosed. In fact, the diagnosis is made when it becomes clinically apparent and in late stage[2].

In 2001, the prevalence of COPD in Africa was estimated 179/100,000 and 301/100,000 in eastern Mediterranean countries. This prevalence was low compared with America and Europe[3].

In Tunisia, prevalence of COPD is estimated as 3.8% (1.1% in women and 6.6% in men) and more than 2000 patients suffer from chronic respiratory failure[4]. In Algeria prevalence of COPD more than 40 years was 125/100,000 people in 1990[2].

COPD affects men more frequently than women, because of less frequent smoking in women. It usually appears after 40 years of age, and increases in frequency with age.

Main risk factors for CRD in MENA are represented by tobacco smoke, second hand tobacco smoke, and other indoor and outdoor air pollutants. In This region, COPD affects people with low socioeconomic status. In fact, it has been demonstrated that smoking is more prevalent in illiterate people[2,5].

Tobacco smoking is established to be the major risk factor of COPD. In Tunisia, according to the study of public health institute, tobacco consumption was reported in 30, 4% of studied population and smoking was 10 times more frequent in men than women[5,6].

In Middle East and Mediterranean countries, smoking in men is varying between 20% in Iran and 63% in Turkey. In Lebanon, more than 30% of women are smokers. In the other countries of the region, smoking concerns less than 10% of women[5].

In MENA, CRD are also a result of environmental factors such as indoor air pollution from biomass fuel, used for cooking and heating, which appears to contribute to COPD in women in MENA countries, but less frequent comparing to African countries[7,8]. An estimated 25-45% of patients with COPD are not smokers but exposed to smoke from biomass, suggesting that exposure to biomass smoke contributes largely in social and economic impact of CRD[8].

In Turkey, the prevalence of COPD was estimated to 18.1% in current smokers more than 40 years of age and was 4.5% among younger smokers; 25.5% of the women and 57.2% of the men were current smokers. Biomass exposure was significantly common among female patients living in rural areas (54.5%). In the genesis of COPD, the relative risk ratio of cigarette smoke was found to be 3.4 and 3.3 times higher than biomass exposure and occupational exposure[9].

In general, the prevalence of COPD in MENA is increasing. In women it is also increasing not only because of exposure to biomass fuels in these countries, but also because of the growing habit of cigarette smoking in women.

### Asthma

The prevalence of asthma increases as communities adopt modern life styles and become urbanized. The European Community Respiratory Health Survey (ECRHS) estimated the prevalence of asthma in adults in Mediterranean countries between 1 and 4%[10]. According to the international Study of Asthma and Allergies in Childhood (ISAAC) in 1998, asthma prevalence was evaluated to 16.5 and 10.7%, respectively, in North Africa and East Mediterranean[11]. However, we admit differences between regions because of their social and cultural habits (Table 1).

**Table 1:** Prevalence of Recent Wheeze and Diagnosed Asthma in Middle East[13]

Location	Wheeze in the Last 12 Months	Physician-Diagnosed Asthma
Cairo	17.4	9.4
Palestine	8.8	9.4
Oman	8.9	20.7
Saudi Arabia	11.2	12.1
Kuwait	16.2	16.8

In Jordan, prevalence of asthma in children is moderate, with no significant difference between the Amman city group and Bedouins (8.8 vs. 9.5%). Authors concluded that there is a 2-fold increase in the prevalence of asthma in Jordan in the last 10 years[12].

In another recent cross-sectional epidemiological survey of asthma, conducted in the Maghreb countries, the overall prevalence of asthma in the general population of Maghreb countries was estimated to be 3.6%, with no relevant difference apparent between the 3 countries (Algeria, Morroco, and Tunisia). This corresponds to a moderate prevalence rate from a worldwide perspective, in accordance to rating of these countries in the most recent GINA burden of asthma report. Highest rates were observed in the densely populated and urbanized regions[14].

## Global Mortality, Dalys, and Economic Costs of Copd in Mena

COPD is a major cause of chronic morbidity and mortality and represents a substantial economic and social burden throughout the world. It is the 5th leading cause of death worldwide and further increases in its prevalence and mortality are expected in the coming decades, being the third cause of death in the world by 2020[1]. In low- and middle-income countries, COPD is also one of the 10 leading causes of death[15].

In Africa, mortality resulting from COPD is globally estimated to 18.1/100,000 in 2001, similar rate (18.3/100,000) was found in East Mediterranean countries[3]. This increase in COPD mortality is because of increases in smoking, particularly in women.

Burden of CRD can also be measured in disability-adjusted life years (DALYs). The world bank estimated that COPD and asthma were responsible for 2.2% of the global burden of disease in East Mediterranean countries in 1999[1]. Worldwide, COPD is expected to move up from the 12th leading cause of DALYs in 1990 to the 5th leading cause in 2020[1,16].

General Cost of CRD in MENA countries remains poorly recognized and difficult to estimate because of lack of data from these countries. However, it is expected that direct costs in COPD are heavy and represent more than half of the total cost, in contrast with asthma in which indirect costs are major.

## Conclusion

Impact of CRD, particularly COPD and asthma in MENA is heavy and needs more actions especially to increase awareness and understanding of CRD and their comorbidities among healthcare workers, healthcare providers, and governments.

This heavy impact is due at primary level to an under diagnosis of CRD, especially COPD, which might be suspected from patient history, clinical symptoms, and confirmed by spirometry.

Effective plans such as education programs and smoking cessation programs are needed to make an early and accurate diagnosis and hence effective management for patients with CRD. National programs for main CRD (COPD and asthma) have to be developed in these countries to standardize their management.

We are still faced with paucity of epidemiological data in MENA countries. The BOLD (Burden of Obstructive Lung Disease) initiative is designed to provide country-specific data on the prevalence, social, and economic burden of COPD[17].
